# What Do We Have to Know about PD-L1 Expression in Prostate Cancer? A Systematic Literature Review. Part 1: Focus on Immunohistochemical Results with Discussion of Pre-Analytical and Interpretation Variables

**DOI:** 10.3390/cells10113166

**Published:** 2021-11-14

**Authors:** Andrea Palicelli, Martina Bonacini, Stefania Croci, Cristina Magi-Galluzzi, Sofia Cañete-Portillo, Alcides Chaux, Alessandra Bisagni, Eleonora Zanetti, Dario De Biase, Beatrice Melli, Francesca Sanguedolce, Moira Ragazzi, Maria Paola Bonasoni, Alessandra Soriano, Stefano Ascani, Maurizio Zizzo, Carolina Castro Ruiz, Antonio De Leo, Guido Giordano, Matteo Landriscina, Giuseppe Carrieri, Luigi Cormio, Daniel M. Berney, Daniel Athanazio, Jatin Gandhi, Alberto Cavazza, Giacomo Santandrea, Alessandro Tafuni, Magda Zanelli

**Affiliations:** 1 Pathology Unit, Azienda USL-IRCCS di Reggio Emilia, 42123 Reggio Emilia, Italy; Alessandra.Bisagni@ausl.re.it (A.B.); Eleonora.Zanetti@ausl.re.it (E.Z.); Moira.Ragazzi@ausl.re.it (M.R.); mariapaola.bonasoni@ausl.re.it (M.P.B.); alberto.cavazza@ausl.re.it (A.C.); giacomo.santandrea@ausl.re.it (G.S.); alessandro.tafuni@ausl.re.it (A.T.); Magda.Zanelli@ausl.re.it (M.Z.); 2 Clinical Immunology, Allergy and Advanced Biotechnologies Unit, Azienda USL-IRCCS di Reggio Emilia, 42123 Reggio Emilia, Italy; Martina.Bonacini@ausl.re.it (M.B.); Stefania.Croci@ausl.re.it (S.C.); 3 Department of Pathology, University of Alabama at Birmingham, Birmingham, AL 35294, USA; cmagigalluzzi@uabmc.edu (C.M.-G.); scaneteportillo@uabmc.edu (S.C.-P.); 4 Department of Scientific Research, School of Postgraduate Studies Norte University, Asunción 1614, Paraguay; alcideschaux@uninorte.edu.py; 5 Department of Pharmacy and Biotechnology (FABIT), University of Bologna, 40126 Bologna, Italy; dario.debiase@unibo.it; 6 Fertility Center, Department of Obstetrics and Gynecology, Azienda USL-IRCCS di Reggio Emilia, 42123 Reggio Emilia, Italy; Beatrice.Melli@ausl.re.it; 7 Clinical and Experimental Medicine PhD Program, University of Modena and Reggio Emilia, 41121 Modena, Italy; Carolina.CastroRuiz@ausl.re.it; 8 Pathology Unit, Policlinico Riuniti, University of Foggia, 71122 Foggia, Italy; francesca.sanguedolce@unifg.it; 9 Department of Pathology, Case Western Reserve University, Cleveland, OH 44106, USA; alessandra.soriano@ausl.re.it; 10 Gastroenterology Division, Azienda USL-IRCCS di Reggio Emilia, 42123 Reggio Emilia, Italy; 11 Pathology Unit, Azienda Ospedaliera Santa Maria di Terni, University of Perugia, 05100 Terni, Italy; s.ascani@aospterni.it; 12 Haematopathology Unit, CREO, Azienda Ospedaliera di Perugia, University of Perugia, 06129 Perugia, Italy; 13 Surgical Oncology Unit, Azienda USL-IRCCS di Reggio Emilia, 42123 Reggio Emilia, Italy; Maurizio.Zizzo@ausl.re.it; 14 Molecular Diagnostic Unit, Azienda USL Bologna, Department of Experimental, Diagnostic and Specialty Medicine, University of Bologna, 40138 Bologna, Italy; antonio.deleo@unibo.it; 15 Medical Oncology Unit, Department of Medical and Surgical Sciences, University of Foggia, 71122 Foggia, Italy; guido.giordano@unifg.it (G.G.); matteo.landriscina@unifg.it (M.L.); 16 Department of Urology and Renal Transplantation, University of Foggia, 71122 Foggia, Italy; giuseppe.carrieri@unifg.it (G.C.); luigi.cormio@unifg.it (L.C.); 17 Barts Cancer Institute, Queen Mary University of London, London EC1M 5PZ, UK; daniel.berney@nhs.net; 18 Federal University of Bahia, Salvador 40110-060, BA, Brazil; dathanazio@gmail.com; 19 Department of Pathology and Laboratory Medicine, University of Washington, Seattle, WA 98195, USA; jgandhi@uw.edu

**Keywords:** PD-L1, prostate, cancer, adenocarcinoma, immunohistochemistry, target-therapy, immunotherapy, checkpoint inhibitors

## Abstract

Immunotherapy targeting the PD-1–PD-L1 axis yielded good results in treating different immunologically ‘‘hot’’ tumors. A phase II study revealed good therapeutic activity of pembrolizumab in selected prostatic carcinoma (PC)-patients. We performed a systematic literature review (PRISMA guidelines), which analyzes the immunohistochemical expression of PD-L1 in human PC samples and highlights the pre-analytical and interpretation variables. Interestingly, 29% acinar PCs, 7% ductal PCs, and 46% neuroendocrine carcinomas/tumors were PD-L1+ on immunohistochemistry. Different scoring methods or cut-off criteria were applied on variable specimen-types, evaluating tumors showing different clinic-pathologic features. The positivity rate of different PD-L1 antibody clones in tumor cells ranged from 3% (SP142) to 50% (ABM4E54), excluding the single case tested for RM-320. The most tested clone was E1L3N, followed by 22C3 (most used for pembrolizumab eligibility), SP263, SP142, and 28-8, which gave the positivity rates of 35%, 11–41% (depending on different scoring systems), 6%, 3%, and 15%, respectively. Other clones were tested in <200 cases. The PD-L1 positivity rate was usually higher in tumors than benign tissues. It was higher in non-tissue microarray specimens (41–50% vs. 15%), as PC cells frequently showed heterogenous or focal PD-L1-staining. PD-L1 was expressed by immune or stromal cells in 12% and 69% cases, respectively. Tumor heterogeneity, inter-institutional preanalytics, and inter-observer interpretation variability may account for result biases.

## 1. Introduction

Prostatic carcinoma (PC) is one of the most common cancers in men, with a 5-year survival rate of 30% at advanced stage [1,2,3,4]. As for other tumors, the implementation of a molecular-based classification and the development of targeted-therapies are increasingly relevant topics of discussion and research, also concerning the possibility to make easy-to-use and cost-effective surrogate biomarkers available worldwide for diagnostic and therapeutic purposes [5].

Programmed death-1 (PD-1) and its ligands (PD-L1 and PD-L2) are type I transmembrane glycoproteins of the CD28/CTLA-4 and B7 families, respectively [6,7]. Microenvironmental conditions may influence the expression of PD-1 by hematopoietic cells (activated T, B, NK cells, and monocytes) and of the two ligands by hematopoietic and non-hematopoietic cells, by modulating their interaction and the subsequent activation of intracellular signaling cascades [6,7].

Immune checkpoint therapy targeting the PD-1–PD-L1 axis has yielded good results in the standard treatment of different immunologically ‘‘hot’’ tumors (such as pulmonary non-small cell carcinoma, renal or bladder cancer, melanoma, etc.). Multiple clinical trials demonstrated sustained survival benefit, sometimes with complete regression of the metastatic disease [6,7,8]. Conversely, immunologically “cold” neoplasms seem relatively resistant to immune checkpoint therapy in some series, as they may show a relatively low somatic mutation frequency and few tumor-infiltrating T cells [6,7,8]. Some studies have been performed on the usually “cold” metastatic castration-resistant prostate cancer (mCRPC), with limited results; treatment doses and schedules could be implemented, especially in selected subgroups of patients, and larger series of patients should be recruited [4,8]. Recently, a phase II study (KEYNOTE-199) seemed to reveal a good therapeutic activity of pembrolizumab (an anti-PD-1 antibody) in monotherapy, and the 2021 United States National Comprehensive Cancer Network (NCCN) guidelines have allowed the use of this drug in selected PC-patients [4,9].

To better clarify the role of PD-L1 expression in PC, we performed a systematic literature review, analyzing the results of human tissue-based studies (immunohistochemical, molecular, etc.), experimental researches (cell lines, mouse models), and clinical trials. As there were many data to discuss, we have split the presentation of our results into different articles, focusing on different sub-topics. In this part, we present the literature data concerning the PD-L1 immunohistochemical expression in human PC samples, highlighting the pre-analytical and interpretation variables.

## 2. Materials and Methods

**Figure 1:**
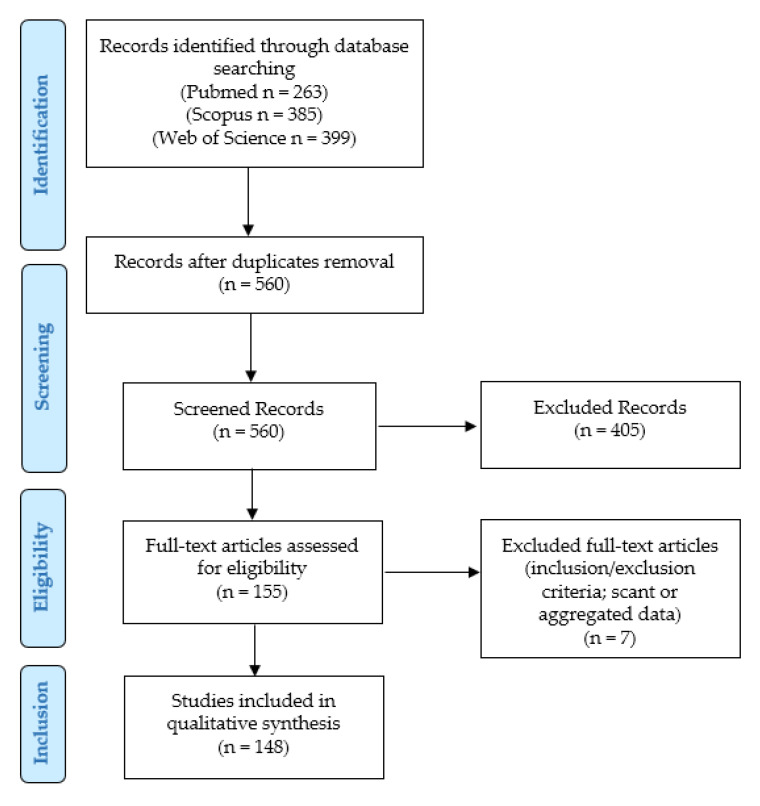
Review of the literature: PRISMA flow-chart.

We conducted our systematic literature review according to the “Preferred Reporting Items for Systematic Reviews and Meta-Analyses” (PRISMA) guidelines (http://www.prisma-statement.org/, accessed on 8 May 2021) (Figure 1).

Our aim was to update and summarize the literature concerning the role of PD-L1 in PC and to report any clinic-pathologic information of the published cases. We tried to answer the following “Population, Intervention, Comparison, Outcomes” (PICO) questions:

- Population: Patients, tumor cell lines, or mouse models included in studies concerning the role of PD-L1 in PC.
- Intervention: Any type of treatment.
- Comparison: No comparisons were expected.
- Outcomes: Patient’s status at the last follow-up (no evidence of disease, alive with disease, dead of disease), response to therapy, biochemical recurrence-free survival, metastasis-free survival, cancer-specific survival, disease-free survival, clinical failure-free survival, overall-survival, and progression-free survival. For experiments on PC cell lines and mouse models: any reported effects on cancer and immune cell migration, proliferation, viability, growth, resistance or response to therapy, cytotoxic or anti-tumor activity, PD-L1 expression, and mice/cell lines survival.

Our research was designed as a retrospective observational study:

- Eligibility/inclusion criteria: experimental studies (tumor cell lines, mouse models) or clinic-pathologic studies on human patients (case series, case reports, clinical trials) concerning the role of PD-L1 in PC.
- Exclusion criteria: non-prostatic tumors; non-carcinomatous histotypes; studies not examining PD-L1; cases with uncertain diagnosis; review articles without new cases.

We searched for (PD-L1 AND (prostate OR prostatic) AND (adenocarcinoma OR adenocarcinomas OR cancer)) in Pubmed (all fields; 263 results; https://pubmed.ncbi.nlm.nih.gov, accessed on 8 May 2021), Web of Science (Topic/Title; 399 results; https://login.webofknowledge.com, accessed on 8 May 2021), and Scopus (Title/Abstract/Keywords; 385 results; https://www.scopus.com/home.uri, accessed on 8 May 2021) databases. No limitations or additional filters were set. The bibliographic research ended on 8 May 2021. After duplicates were excluded, 560 records underwent first-step screening by two independent reviewers, who checked the titles and abstracts of the articles to verify the satisfaction of the eligibility/inclusion criteria. They excluded clearly irrelevant studies, allowing articles of doubtful relevance to proceed to the following step, in order to avoid the potential missing of pertinent papers. The full texts of the 155 eligible articles were retrieved, and read by two other authors: (1) to look for additional relevant references; and (2) to confirm the satisfaction of the inclusion criteria. After their evaluation, seven papers were excluded, as they were unfit according to the inclusion criteria, or because they presented scant or aggregated data. Two other authors checked the extracted data, and 148 articles were finally included in our study [8,9,10,11,12,13,14,15,16,17,18,19,20,21,22,23,24,25,26,27,28,29,30,31,32,33,34,35,36,37,38,39,40,41,42,43,44,45,46,47,48,49,50,51,52,53,54,55,56,57,58,59,60,61,62,63,64,65,66,67,68,69,70,71,72,73,74,75,76,77,78,79,80,81,82,83,84,85,86,87,88,89,90,91,92,93,94,95,96,97,98,99,100,101,102,103,104,105,106,107,108,109,110,111,112,113,114,115,116,117,118,119,120,121,122,123,124,125,126,127,128,129,130,131,132,133,134,135,136,137,138,139,140,141,142,143,144,145,146,147,148,149,150,151,152,153,154,155].

Data collection was study-related (authors and year of study publication) and case-related (PD-L1 expression; tumor stage at presentation; Grade Group/Gleason score; type of specimen; treatment; test methods; and results of PD-L1 expression, follow-up, outcomes, and experiment type).

Statistical analysis: the collected data were reported as continuous or categorical variables. Categorical variables were analyzed by frequencies and percentages; continuous variables were summarized by ranges, mean, and median values. Time-to-recurrence was the time from primary treatment to disease recurrence. The survival status was the time from primary treatment to the last follow-up.

## 3. Results

### 3.1. PD-L1 Immunohistochemical Expression in Tumor Tissue: Benign Glands vs. Adenocarcinoma

Haffner et al. [66] found that benign atrophic glands (especially those associated with inflammation) were frequently PD-L1-positive. Conversely, PD-L1 expression was rarely reported in benign prostatic tissue or hyperplasia in the remaining studies: Sharma et al., 1/201 (0.5%) cases [27,35]; Richter et al., 1/24 (4%) cases (focal expression) [55]; and Sun et al., 0/30 (0%) cases [13].

In some studies, PD-L1 expression was significantly higher in PCs of all stages compared to benign prostatic hyperplasia or normal prostatic tissue (Wagle et al., *p* < 0.0001 [28]; Sharma et al., *p* < 0.001 [35]). Richter et al. also found focal PD-L1 staining in <1% cells of a prostatic intraepithelial neoplasia [55].

Scimeca et al. described a significant increase in the number of PD-L1+ (*p* = 0.0047) and PTX3+ (*p* < 0.0001) cells in PCs (vs. benign lesions) [48]. Hahn et al. [57] reported that the immune infiltrates were higher in PCs than benign prostatic tissues (lymphocytes *p* < 0.001, macrophages *p* = 0.01).

### 3.2. PD-L1 Immunohistochemical Expression in Prostate Cancer

Table 1 summarizes the results of the studies concerning the immunohistochemical expression of PD-L1 in tumor cells of acinar PCs. Further details of the cases are provided in Supplementary Materials Table S1.

1369/4708 (29%) PCs were overall classified as positive but different antibody clones, scoring systems, and/or cut-offs for positivity were used, with a range as wide as 0–100% in single studies. 687/2676 (26%) cases were considered PD-L1-positive if at least ≥1% of tumor cells were stained [8,13,27,29,35,36,37,38,39,41,42,43,54,62,66,67,74,75,77,78,79,84], while 93/1062 (9%) PCs were counted as positive for ≥5% of stained tumor cells [17,39,43,57,66,94,98,99,100,120]. 9/523 (2%) cases expressed PD-L1 on >50% of tumor cells [66,92], while Wang et al. used a cut-off of ≥25% (0/21, 0%) [61]. The remaining studies applied different scoring criteria, also including tumor proportion score (TPS) (9/51 PD-L1+ PCs, 18%) [59] or combined positive score (CPS ≥ 1: 362/464 cases, 78%) [9,154].

In other series, it was unclear how many PCs were tested or resulted PD-L1+. In human tumors, mutations in the PD-L1 C-tail are mutually exclusive with mutations in the substrate-interacting MATH domain of the “Speckle-type BTB/POZ protein” (SPOP) [73]. In the series of Zhang et al. (*n* = 97), 80% of 15 *SPOP^mutant^* tumors exhibited strong PD-L1 staining (clone 29E.12B1), compared to only 10% of 82 *SPOP^wild-type^* PCs. SPOP-deficient PCs correlated with increased PD-L1 expression and a decreased number of tumor infiltrating lymphocytes (TILs) [73]. In another large series [47], PD-L1 was expressed at lower levels than PD-L2 across all 9393 radical prostatectomy samples (tissue microarrays, TMA) (*p* < 0.001). The authors obtained data from six previously published retrospective cohorts (*n* = 1567) and one prospective cohort (*n* = 47826), but the method of PD-L1 testing and scoring was not fully disclosed. Using a median value cut-off, other researchers [89,93] found that PD-L1 was highly expressed in 486/820 (59%) PCs of their series, positively correlating with elevated (>median value) Ki-67 index and AR expression (*p* < 0.001).

Calagua et al. [75] reported 18/130 (14%) PD-L1+ hormone-naïve PCs associated with increased CD3+, CD8+, and PD-1+ TILs. Tumor cells showed three patterns of PD-L1 positivity:

1. “scattered” pattern (10 cases, 56%) (small clusters);
2. “nodular” pattern (7 cases, 39%) (distinct nodules): the PD-L1^high^ and PD-L1^low^ components showed consistently concordant ERG status but variable PTEN staining patterns;
3. “interface” pattern (1 case, 5%): positive tumor cells were enriched at the tumor–stroma interface bordering the leading edge of a tumor nodule.

In their series, all the three examined PCs treated with neoadjuvant abiraterone acetate, prednisone and leuprolide revealed the “scattered” pattern of PD-L1 staining on tumor cells [75].

The positivity rate of different PD-L1 antibody clones in PC cells ranged from 3% (SP142) to 50% (ABM4E54), excluding the single case tested for RM-320. The most tested clone was E1L3N, followed by 22C3, SP263, SP142, and 28-8. The positivity rates were 35%, 11–41% (depending on different scoring systems), 6%, 3%, and 15%, respectively. The other clones were tested in less than 200 cases (Table 2).

TILs were variably present in histologic PC specimens; few studies reported a quantification and characterization of the inflammatory infiltrate. Some authors described 156/396 (39%) cases showing sparse intratumoral PD-1+ lymphocytes (few intraepithelial) [42,79]. Iacovelli et al. [39] found macrophages and CD3+ T-lymphocytes in 10% of PD-L1− cases and in 21% of PD-L1+ PCs, while neutrophils were rarely observed (two cases) and only 1/5 PCs showed scattered PD-L1+ dendritic cells. Petitprez et al. [77] reported a significant association of PD-L1 expression with CD8+ T cell density (*p* = 0.040), but not with CD20+ B-cell density. In another study [50], FOXP3+, CD3+, and CD8+ T cells were observed in 74.6% (47/63), 98.4% (62/63), and 100% (61/61) of the cases, respectively. Finally, Massari et al. found TILs in 11/16 (69%) cases [92].

PD-L1+ immune cells (mainly TILs) were identified in 76/634 (12%) cases [21,27,32,35,41,51,57,67,74,90]. Sater et al. [34] reported that neoadjuvant prostate specific-antigen (PSA)-targeted vaccination with PROSTVAC prior to radical prostatectomy enhanced T cell infiltration into the tumor microenvironment of PCs. PD-L1 expression (by immunofluorescence) was focal in the immune (macrophage-like) cells, and the PD-L1+ status correlated to higher post/pre-ratio of immune-cell infiltrates. CD4+ T cells were significantly increased in post-vaccination radical prostatectomy specimens compared to baseline biopsies by non-compartmentalized analysis. By compartmentalized analysis, an increase in CD4+ T cells at the tumor infiltrative margins, as well as of CD8+ T cells at the tumor core, was noted in post-vaccination specimens rather than in baseline biopsies. In addition, 13/25 (52%) patients developed peripheral T cell responses to any of the three tested tumor-associated antigens (PSA, brachyury, MUC-1).

PD-L1 was expressed by stromal cells in 349/505 (69%) cases [42,50,62,79]. Mo et al. [50] found that 69/73 (94.5%) regionally localized PCs (RLPCs) and 2/7 (29%) CRPCs were positive for PD-L1 in tumor-associated stroma (nerve branches), supported by colocalization with axonal marker PGP9.5. The density of PD-L1+ tumor-associated nerves inversely correlated to that of CD8+ TILs. In another study [42,79], the expression of PD-L1+ intratumoral stromal cells (267/402, 66%) was significantly associated with PD-L1+ tumor cells (*p* < 0.001), showing a weak correlation with intratumoral PD-1+ lymphocytes (*p* < 0.001). Li et al. [44] also reported that stroma and TILs partially expressed PD-L1: PD-1 was mainly positive at the membrane of the stromal infiltrating lymphocytes (31/127 cases, 24.4%).

### 3.3. PD-L1 Immunohistochemical Expression in Different Specimen-Types of Prostatic Adenocarcinoma

PD-L1 expression was investigated in different types of specimens, including biopsies, radical prostatectomies (RPs), transurethral prostatic resections (TURPs), metastases, and/or material retrieved from autopsies. Finally, TMAs were used by some authors [27,29,35,39,41,53,66,67,74,78,83,92,112,120]. This method allowed the evaluation of larger series, but reduced the number of tumor cells evaluable for PD-L1, which usually showed heterogeneous or focal expression. In many series, the tested specimen-type was not specified. Other studies analyzed different samples, globally reporting the positivity rate of PD-L1 without highlighting the data for each specimen type. Finally, some articles just described the results of the statistical comparisons among various subgroups, without providing detailed information for each of them (Table S1). Haffner et al. [66] reported no variation of the PD-L1 positivity rate in their TMA series (*n* = 508) despite including different samples (core biopsies, surgical resections, post-mortem rapid autopsies), while Shaw et al. found a significantly higher PD-L1 expression in resection specimens compared to biopsies (*p* = 0.045; *n* = 63) [36].

When data were available and analyzable, the PD-L1 positivity rate of non-TMA specimens was 50% (240/483) for biopsies [9,11,12,21,32,62,90] and 41% (587/1427) for RPs [18,38,42,43,44,57,61,75,79,94]. In TMA series (variably including RPs, biopsies, TURPs, metastases, autopsies), the expression rate was lower (258/1728, 15%) [27,29,35,39,41,53,66,67,74,78,83,92,112,120]. Considering only RPs, PD-L1 was more frequently positive in non-TMA specimens (587/1427, 41%) than in TMA-RPs (130/762, 17%) (total: 717/2189, 33%) [18,27,29,35,38,42,43,44,57,61,67,75,79,92,94,112,120].

### 3.4. PD-L1 Immunohistochemical Expression in Rare PC-Histotypes

Previous data referred to the acinar histotype of PC, which is by far the most common [1]. Only few studies focused on PD-L1 expression in rare PC-histotypes:

- **Ductal carcinoma** (8/119 PD-L1+ cases, 7%) [36,41,66,78].

In all the studies, positivity was assessed for ≥1% of tumor cells stained for PD-L1. The positivity rate ranged from 3% to 17% among various series. It was higher for clone SP263 (5/58, 9%: Lindh et al., 1/34, 3% [41]; Haffner et al., 4/24, 17% [66]), while clone E1L3N resulted positive only in 2/34 (6%) cases [78], and an unspecified clone was used by Shaw et al. (1/27 PD-L1+ cases, 4%) [36]. In addition, Hashimoto et al. reported 2/110 (2%) PD-L1+ cases: the series included 105 acinar, 4 ductal, and 1 signet ring PC, but it was unclear which histotypes resulted positive [85]. Lindh et al. [41] found 10/34 (29%) ductal adenocarcinomas showing PD-L1+ tumor-infiltrating cells (positivity in 1–20% of cells), including the case with PD-L1+ tumor cells, while PD-L2 was negative in both tumor cells and tumor-infiltrating immune cells in all cases. Ductal adenocarcinomas showed a significantly increased number of CD8+ lymphocytes compared to acinar adenocarcinomas (*p* = 0.04).

- **Neuroendocrine carcinomas** (25/48 PD-L1+ cases, 46%).

In the abstract of Sun et al. [111], PD-L1 was rarely expressed in primary acinar PCs, while increased rates of PD-L1 positivity were observed in prostatic small cell neuroendocrine carcinomas (SCNCs); however, the number of tested samples was unclear. In a subsequent paper [13], PD-L1 positivity was observed only in the SCNC group (*n* = 16) (which were N-cadherin-positive), while other samples seemed not to express PD-L1 (30 benign prostates, 15 “low level” adenocarcinomas, 15 “high level” adenocarcinomas, 18 CRPCs). “Low”- and “high”-level adenocarcinomas were not defined, while the PD-L1 clone and scoring systems were unclear.

Von Hardenberg et al. [52] tested a series including 7 adenocarcinomas with neuroendocrine differentiation (ND), 20 SCNCs, 2 well-differentiated neuroendocrine tumors (NET), and 10 adenocarcinomas. PD-L1 positivity (>1% of PD-L1+ tumor cells; clone E1L3N) was identified in 5/24 (20.8%) cases, but it was unclear which histotypes resulted positive. High PD-L1 expression was observed in 4/11 (36.4%) specimens. TILs (≥10%) were found in 9/24 (37.5%) cases. All the PCs were negative for clone 22C3. On gene expression analysis, positive PD-L1 status was observed in hot spots of 11 (61.1%) cases (4/5 adenocarcinomas with ND, 6/12 SCNC, 1/1 NET, and 4/4 adenocarcinoma). In addition, the PD-L1 mRNA expression in hot spots or TILs had no prognostic significance.

The neuroendocrine PC of Massari et al. revealed strong expression of CD3, PD-1, and PD-L1 (anti PD-L1 clone, Sino Biological, Eschborn, Germany; weak/strong positivity in >50% tumor cells) [92].

Finally, Haffner et al. found 3/7 (42.9%) PD-L1+ SCNCs (clone SP263) [66].

- **Carcinosarcoma** (1/1, 100%; clone SP142).

In the abstract of Sun et al. [111], PD-L1 was rarely expressed in primary acinar PCs, PCs, Salvi et al. [60] reported a prostatic carcinosarcoma in which the epithelial component was PD-L1-positive (“high” expression; Gleason Score 9, 4+5, Grade Group 5), while the sarcomatous areas were negative.

### 3.5. Evaluation of PD-L1 Expression Density in Tumor Tissue

Few studies [15,25,26,28,31,34,40,48] (Table S2) evaluated the PD-L1 expression density by counting the number of PD-L1+ tumor and/or immune cells in variably analyzed areas. Different methods of analysis and scoring were used. Moreover, the difference in PD-L1 expression (percentage of positive cells, intensity of staining) between tumor and immune cells groups was frequently unclear. In fact, the different cell types were cumulatively counted in some articles, while the analyzed cell type was unclear in other papers; thus, it was sometimes difficult to understand which cells resulted positive for PD-L1.

Ryan et al. [26] found that PD-L1 staining primarily occurred in tertiary lymphoid structures of high-risk PCs, while a non-significant decrease in the mean PD-L1 density was observed in rituximab-treated tissues (*n* = 8) vs. control samples (*n* = 11) (*p* = 0.36).

Scimeca et al. reported a significant increase in the number of PD-L1+ (*p* = 0.0047) and PTX3+ (*p* < 0.0001) cells in PCs (*n* = 50) (vs. benign lesions). PD-L1+ PCs showed a decrease in PD-1+ lymphocytes and tumor-infiltrating macrophages (mainly M2 subpopulation), while PTX3 expression inversely correlated to the number of PD-L1+ PC cells [48].

Kazantseva et al. [40] generated three different groups of PCs (*n* = 122) by clustering analysis. Compared to the other two groups, group A (43 cases, 35%) had significantly more PD-L1+ cells (*p* = 0.01), higher *Δ133TP53* and *TP53β*, a significantly higher number of infiltrating T cells (CD3+, CD4+, and/or CD8+) and CD20+ B cells, as well as an increased number of PD-1+ T cells. Group A also showed significantly higher number of infiltrating CD163+ macrophages (compared to Group C), and higher Ki67 index (compared to Group B). Group C had less perineural invasion than Group B and lower Gleason Score than Groups A/B.

In another study [28], high tumor fusion burden (number of fusions/10,000 genes) correlated to high immune infiltration, PD-L1 expression on immune cells (negative on tumor cells), and immune signatures of T cells and M1 macrophages activation; conversely, it inversely correlated to immune suppressive signatures. Only late metastatic samples (*n* = 3) showed ≥10% of PD-L1 + inflammatory cells.

Ihle et al. reported higher PD-L1 expression in blastic (*n* = 5) than in lytic bone PC metastases (*n* = 10) [31].

Brady et al. [15] analyzed 27 mCRPC with digital spatial gene expression profiling. PD-L1 protein levels were not detectable above the background in any tumor or tumor regions of interest (ROI), and CTLA4/PD-1 expression were below measurable levels in >90% of ROI.

By using fluorescent immunohistochemistry on TMAs, Vicier et al. [25] analyzed the total density of PD-L1 positivity in 109 PCs. CD8 ^low^ and/or PD-L1^high^ expression was associated with shorter time to biochemical recurrence and metastasis-free survival (median 10.8 vs. 18.4 years) compared to CD8^high^/PD-L1^low^ cases. However, this association was not significant on multivariate analysis.

## 4. Discussion

The implementation of a molecular-based classification and the development of targeted therapies are increasingly relevant topics of discussion and research in numerous tumors, especially regarding the search for cost-effective, surrogate diagnostic, prognostic, and treatment-predictive biomarkers [1,2,3,4,5,156,157,158,159,160,161,162,163,164,165]. Promising pre-clinical models, early clinical results, and some case reports provided proof of concept for considering selected mCRPC patients as suitable candidates for treatment with immune checkpoint blockade [4,8,9,10,11,12,13,14,15,16,17,18,19,20,21,22,23,24,25,26,27,28,29,30,31,32,33,34,35,36,37,38,39,40,41,42,43,44,45,46,47,48,49,50,51,52,53,54,55,56,57,58,59,60,61,62,63,64,65,66,67,68,69,70,71,72,73,74,75,76,77,78,79,80,81,82,83,84,85,86,87,88,89,90,91,92,93,94,95,96,97,98,99,100,101,102,103,104,105,106,107,108,109,110,111,112,113,114,115,116,117,118,119,120,121,122,123,124,125,126,127,128,129,130,131,132,133,134,135,136,137,138,139,140,141,142,143,144,145,146,147,148,149,150,151,152,153,154,155]. Moreover, PC is one of the first adult solid tumors demonstrating to benefit from a cancer vaccine, further supporting the potential usefulness for immunotherapy. The 2021 NCCN guidelines have allowed immunotherapy in men with asymptomatic or minimally symptomatic mCRPCs [4]. Sipuleucel-T is a dentritic cell-based autologous cancer vaccine approved by the Federal Drug Administration (FDA) and the NCCN guidelines for treatment of mCRPCs. However, the survival benefit and efficacy are modest, and further progress is required in understanding which patients may benefit from treatment [4].

As better delineated in other parts of our review (see the end of the Discussion section), experimental studies revealed that the intracellular ERK/MEK, Akt-mTOR, NF-kB, WNT, and JAK/STAT pathways are involved in PD-L1 upregulation in PC cells. PD-L1 interacts with its receptor PD-1, which can be expressed by inflammatory cells (T, B, NK, myeloid, etc.), favoring the process of immune evasion by tumor cells. The PD-1–PD-L1 interaction inhibits the T cell receptor signaling, suppressing the activity and proliferation of cytotoxic TILs, and promoting the immunosuppressive effects of regulatory T cells. Moreover, the PD-1–PD-L1 axis influences all the components of the tumor microenvironment, regulating the inhibition of NK cells (which can kill tumor cells directly), the secretion of cytokines, and the immunosuppressive effects of tumor-associated macrophages, dendritic cells, and myeloid-derived suppressive cells.

Blocking the PD-1–PD-L1 signaling can prevent tumor immune escape, and increase the anti-tumor activity of immune cells, despite some clinical trials reported limited or controversial benefits (especially if including unselected patients) [4,108,123,126]. Pembrolizumab can be administered to men with docetaxel- or hormone-resistant mCRPCs showing high microsatellite instability or mismatch repair system deficiency (MSI-H/MMR-d), or tumor mutation burden >10 mutations/Mb [4]. However, MSI-H/MMR-d PCs occur with a low frequency, and the immunotherapy administration is still limited in the routine management of PC-patients [4]. Moreover, the MMR-d/MSI-H status does not necessarily correlate to the response to immunotherapy [46,59].

The NCCN guidelines do not report the need of a histochemical evaluation of PD-L1 in PCs and there are no clear indications concerning the PD-L1 clone to use for evaluating PC samples [4]. So, PD-L1 expression may not be useful alone for patient selection in PCs. The focal PD-L1 positivity may not predict the responsiveness of the tumor, and clinical studies reported PD-L1+ PCs resistant to immunotherapy, as well as PD-L1 cases responding to treatment. Moreover, in some clinical trials, not all the samples were tested for PD-L1 or it was unclear how many cases were analyzed by immunohistochemistry [9,21,24,32,62,90,116]. Other immune checkpoints or targets that may be clinically relevant in PC (VISTA, PARP, etc.) could help for the selection of patients. Indeed, reliable biomarkers should guide the selection of patients for clinical trials, monitor treatment efficacy, and allow the development of more effective combinatorial strategies. Multiple targeting may improve the immunogenicity of the “cold” PC tumor cells. In other parts of our review, we will discuss the results of clinical trials testing anti-PD-1–PD-L1 drugs, as of studies investigating both the PD-L1 and MMR-d/MSI-H status.

Our systematic review on the expression of PD-L1 in PC highlights a high heterogeneity across studies [8,9,10,11,12,13,14,15,16,17,18,19,20,21,22,23,24,25,26,27,28,29,30,31,32,33,34,35,36,37,38,39,40,41,42,43,44,45,46,47,48,49,50,51,52,53,54,55,56,57,58,59,60,61,62,63,64,65,66,67,68,69,70,71,72,73,74,75,76,77,78,79,80,81,82,83,84,85,86,87,88,89,90,91,92,93,94,95,96,97,98,99,100,101,102,103,104,105,106,107,108,109,110,111,112,113,114,115,116,117,118,119,120,121,122,123,124,125,126,127,128,129,130,131,132,133,134,135,136,137,138,139,140,141,142,143,144,145,146,147,148,149,150,151,152,153,154,155], causing limitations to our analysis.

However, these results provide ground to discuss various issues related to the assessment methodologies and processes of PD-L1 expression by PC in clinical practice.

**Figure 2:**
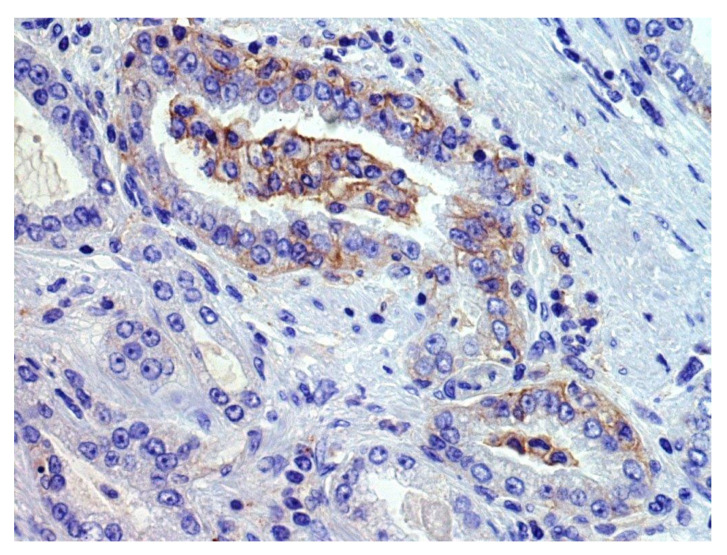
An example of focal membranous PD-L1 immunohistochemical staining (previously unpublished, original photo; magnification 400×, Clone 22C3, Dako).

We found that 29% of acinar PCs, 7% of ductal PCs, and 46% of neuroendocrine carcinomas or tumors were classified as PD-L1-positive by immunohistochemical analysis on tumor tissue, despite these results may be influenced by some selection biases [8,9,11,12,13,17,21,27,29,32,35,36,37,38,39,41,42,43,44,50,51,52,53,54,55,57,59,60,61,62,66,67,74,75,77,78,79,83,84,85,89,90,92,93,94,98,99,100,111,112,120,154]. In fact, when reported, different scoring systems (CPS, TPS, semiquantitive analysis, percentage of positive cells × staining intensity, etc.) and cut-offs for positivity (<1%, ≥1%, ≥5%, ≥25%, ≥50%, ≥ median value, etc.) were applied. Various types of specimens were tested (radical prostatectomies, transurethral resections of the prostate, biopsies, autopsy samples; primary vs. metastatic tumors), revealing variable positivity rates also among the different sampled sites of a single patient. Moreover, PCs showing different clinic-pathologic features (Grade Group, stage, treatment type, etc.) were tested, but the potential association of PD-L1 expression with clinic-pathologic features was not always clearly investigated. In addition, various PD-L1 antibody clones were rarely simultaneously tested on the same series. Considering the heterogenous or focal expression of PD-L1 by PC cells (Figure 2), the use of TMA reduced the number of evaluable cells, probably causing false-negative results in some cases; conversely, some trials included only PD-L1+ cases, causing a selection bias for the abovementioned positivity rates [154].

Inter-observer variability in the interpretation of PD-L1 immunohistochemical staining may frequently occur, but it could be decreased by the support of inter-institutional teams of experts, training, and quality controls [166]. An issue in assessing PD-L1 expression in PC samples may be represented by the concomitant staining of both immune (lymphocytes, plasma cells, granulocytes, mast cells) and stromal cells, which may be a confounding factor, especially when both the tumor and immune cell compartments need to be evaluated in a composite score. Our results showed that PD-L1+ immune cells (mainly TILs) were found in 12% cases, and that PD-L1 was expressed by stromal cells in as many as 69% cases. Moreover, T-helper lymphocytes were significantly increased in post-vaccination radical prostatectomy specimens compared to baseline biopsies in one study [34].

Plasticity of the immune response and the effects of the different tissue microenvironment components were not simultaneously evaluated in most of the PD-L1 testing series. The studies were usually retrospective, rarely providing multicenter validation cohorts.

Several clones of the PD-L1 antibody are currently feasible for immunohistochemical testing and are used in clinic-pathologic studies and clinical trials to identify patients that can benefit from immunotherapy. Few clones have been approved as companion or complementary diagnostics in various tumors [166]. However, there are different PD-L1 assays, each specific to a therapeutic drug for disparate tumors, without a common standard [166]. Moreover, various scoring systems are applied to different tumor types and indications. Finally, some issues are difficult to solve, including tumor heterogeneity of PD-L1 expression, inter-institutional preanalytics, and inter-observer variability in interpretation of the results [156,162,166].

Clone 22C3 is typically tested in non-small cell lung cancer (NSCLC) or gastro-esophageal adenocarcinoma for pembrolizumab treatment, clone 28-8 for nivolumab therapy in melanoma, clone SP263 for durvalumab administration in NSCLC, and clone SP142 for atezolizumab use in triple negative breast cancer [166]. Discrepancies in indications are also present among different countries. In the United States, the FDA restricted the application of pembrolizumab for advanced NSCLCs tested using the DAKO 22C3 pharmDx assay, while the DAKO platform is not as widely used in Europe, and the European Medicines Agency (EMA) did not demand a mandatory specific PD-L1 immunohistochemical platform or clone [166]. As regards to genito-urinary tumors, clones SP263, SP142, 28-8, and 22C3 have been approved as assays to identify urothelial carcinoma patients that are most likely to benefit from various immunomodulants. To select eligible patients, each clone should be evaluated on the basis of specific score system and/or cut-off criteria (SSCC), according to current guidelines based on the results from large clinical trials: Atezolizumab (SP142; SSCC: ≥5% of positive immune cells); avelumab, durvalumab, and tislelizumab (SP263; SSCC: ≥25% of positive tumor or immune cells); nivolumab (28-8; SSCC: ≥1% of positive tumor cells); and pembrolizumab (22C3; CPS score) [167,168]. Such discrepancies in the recommended algorithms of evaluation reveal defective harmonization of PD-L1 immunohistochemical testing.

As we previously said, the NCCN guidelines do not report the need of a histochemical evaluation of PD-L1 in PCs and there are no clear indications concerning the PD-L1 clone to use for evaluating PC samples [4]. In the studies analyzed in our review, the most frequently tested clone was E1L3N, followed by 22C3, SP263, SP142, and 28-8. Other clones were tested in less than 200 cases (Table 2). Of them, E1L3N, SP263, and SP142 target epitopes in the cytoplasmic domain, which is identical for SP263 and SP142. Conversely, 22C3, 28-8, and 5H1 target distinct epitopes within the extracellular domain [169]. Recently, Lawson et al. [169] performed linear and discontinuous epitope mapping of the binding sites of PD-L1 antibodies by peptide array. Their analysis revealed that immunohistochemical results are not significantly altered by differences in epitope binding.

Based on the results of our review, the positivity rate of different PD-L1 antibody clones in tumor cells ranged from 3% (SP142) to 50% (ABM4E54), excluding the single case tested for RM-320. The positivity rate of clone 22C3 (the most used to assess eligibility for administration of pembrolizumab) was 11% after excluding cases with a CPS score of ≥1 (as it was unclear if tumor cells resulted positive), 41% after including those cases, and 78% only considering the cases scored as CPS ≥1 [9,12,13,21,27,32,35,51,77,80,83,90,154].

In recent years, some evidence from the literature has suggested that substantial concordance in both staining results [170,171] and clinical outcomes [170,172] was yielded by the three FDA-approved clones (22C3, 28-8, and SP263) when tested on NSCLC specimens. Limited similar data are also available for urothelial carcinoma [173].

However, the performance of different diagnostic anti PD-L1 antibody clones in other tumors (such as head and neck squamous cell carcinoma) is less robust and interchangeable [166]. In our analysis, the staining rates of these three clones were 11–41%, 15%, and 6%, respectively; these results (with the abovementioned caveats of clone 22C3) may seem lower than for other clones, which, in turn, yielded ≥35% rates (E1L3N: 35%, PA5-18337: 39%; ABM4E54: 50%; 5H1: 46%; anti-PD-L1: 47%) with the exception of SP142 (3%). Calagua et al. [75] found that E1L3N and SP142 clones demonstrated excellent concordance (correlation coefficient *r* = 0.90; *n* = 174), while antibody 405.9A11 showed the lowest sensitivity (suboptimal).

Some studies have reported details on the staining pattern and/or intensity of each antibody, although substantial differences have been found in both cases, potentially affecting the final PD-L1 scoring [167]. Calagua et al. [75] showed three patterns of PD-L1 expression in PCs but further analysis was not performed in subsequent papers. In their study, clone E1L3N consistently stained nerve fibers, unlike SP142 or SP263 [75]. In contrast, SP142 showed non-specific staining in luminal secretions and in the cytoplasm of benign and malignant glands (absent with E1L3N). In another paper [74], clone SP263 showed a stronger membranous staining in tumor cells than clone E1L3N, but only five PD-L1 positive cases were found.

Features intrinsic to the tissue sample should be taken into account in assessing PD-L1 expression, including biological intratumor heterogeneity (as abovementioned) and pre-analytical variables, which may affect the final results [166]. While the former is largely unpredictable, the latter should be standardized by following strict laboratory protocols [167]. However, differences in staining concordance and interpretation may occur among various institutes [166,174]. Recently, Angels et al. [175] reported a significant rate of suboptimal PD-L1 immunostaining (clones SP142 and SP263) in tonsillectomy specimens exposed to more than 72 h in formaldehyde. As for other tumor tissues, overfixation may influence the staining results. Standardization of the type of platform and testing method by using proper positive and negative controls are of pivotal importance at this regard. It is advisable to provide all the technical information, along with the results and the used scoring system, in the final pathological report [167].

Finally, systematic literature reviews (SLRs) and meta-analyses have become increasingly important in health care as clinicians read SLRs to keep themselves up-to-date. Moreover, SLRs are often a starting point for developing clinical practice guidelines or further studies/trials, while granting agencies may require data of SLRs to justify research financial supports. For these reasons, medical journals frequently ask contributing authors to conduct their SLRs according to the PRISMA (http://www.prisma-statement.org/; accessed on 8 May 2021) guidelines (or similar), which include an evidence-based minimum set of items for reporting and are useful for the critical evaluation of the submitted manuscripts [176,177,178,179,180,181,182,183,184,185,186,187,188,189,190,191,192,193,194,195,196,197,198,199,200,201,202,203,204,205,206]. So, we have conducted our SLR according to these guidelines, as previously described in various topics/contexts in which they are applicable and could provide relevant benefits in dissemination of knowledge [176,177,178,179,180,181,182,183,184,185,186,187,188,189,190,191,192,193,194,195,196,197,198,199,200,201,202,203,204,205,206]. To better examine the multiple interesting facets of the role of PD-L1 in PCs, we have divided the discussion of our results into different articles. In the other papers, the readers will find further information on sub-topics not discussed in the present article, including correlations of PD-L1 expression with clinic-pathologic features in PC patients; genetic and epigenetic regulation of PD-L1; PD-L1 intracellular signaling pathways in PC and influence of the tumor microenvironment; data of pre-clinical studies (cell lines, mouse models) about the effects of experimental treatments on PD-L1 expression in PC cells; investigated correlations of PD-L1 expression with the status of mismatch repair system, *BRCA*, *PTEN,* and other main genes in PC; PD-L1 expression in liquid biopsy samples; and information of clinical trials [207,208,209,210].

## 5. Conclusions

In this part of our review, we present some results that could be helpful for the design of further studies or trials. We have summarized the data retrieved from our SLR, which was performed according to the PRISMA guidelines, by searching in multiple databases. Immunohistochemical PD-L1 positivity was usually focal in the tumor cells of about 29% acinar PCs, 7% ductal PCs, and 46% neuroendocrine carcinomas/tumors, despite some limitations. Most of the available data were related to the acinar type of PC. Additional studies are required, especially for rare PC histotypes, such as ductal and neuroendocrine carcinomas, with the latter showing a higher PD-L1 positivity rate.

The assessment of PD-L1 immunohistochemical expression and the resulting positivity rate are influenced by various factors, including different antibody clones, scoring systems and cut-off criteria for positivity, tumor heterogeneity, sample type (primary vs. metastatic tumors, RPs, biopsies, autopsies, TMAs), inter-observer variability in interpretation, and other pre-analytical variables (fixation, etc.). This information was sometimes lacking or unclear, also in some clinical trials. Further studies are required to clarify the role (and the need) of PD-L1 immunohistochemical testing in PCs, and the eventual difference in clinical outcome among cases with limited vs. diffuse expression, also paying attention to differences in scoring systems. Indeed, in the analyzed studies, only tumor cells were usually evaluated, while the CPS score (also including immune cells in the count) was rarely applied.

The 2021 NCCN guidelines have allowed pembrolizumab administration in selected MSI-H/MMR-d mCRPC patients, progressing through prior docetaxel and/or novel hormone therapy [4]. However, these guidelines do not currently report the need of an immunohistochemical evaluation of PD-L1 in PC, and there are no clear indications concerning the PD-L1 clone to use for evaluating PC samples. Reasons for this lack of indication could include the following: (1) PC is a “cold” tumor sometimes showing limited intratumoral inflammation and response to immunotherapy; (2) some immunohistochemically PD-L1+ tumors may not respond to immunotherapy, while PD-L1 cases can respond; (3) the frequently focal PD-L1 immunohistochemical expression (positivity was usually assessed for ≥1% of stained cells) could not be a reliable predictor of the tumor responsiveness to immunotherapy; (4) it is unclear which is the best sample to test, as the positivity rate varies among different types of specimen; (5) data about the immunohistochemically tested cases (number of analyzed PCs; correlation with some clinic-pathologic features; and the abovementioned pre-clinical and interpretation variables) were occasionally missing or limited in some clinical studies/trials (sometimes lacking multicenter validation cohorts).

Future researches should clearly provide details of these pre-analytical variables, which can also be taken into consideration in making comparisons between the results of the different analyzed groups. However, PD-L1 immunohistochemical expression could be confirmed not to be useful (at least alone) to select PC patients for the inclusion in clinical trials. Other immune checkpoints or targets that may be clinically relevant in PCs should be investigated, supporting the trial selection and/or allowing the development of more effective combinatorial strategies for treatment. The intracellular ERK/MEK, Akt-mTOR, NF-kB, WNT, and JAK/STAT pathways are involved in PD-L1 upregulation in PC cells. An estimate of expression of PD-L1 and of markers involved in these (and other) signaling pathways may be combined in future clinical studies. Multiple targeting may improve the immunogenicity of PC.

## Figures and Tables

**Table 1:** PD-L1 immunohistochemical expression in tumor cells of prostatic acinar adenocarcinomas.

Authors	Positivity Rate	Antibody Clone	Scoring Criteria for Positivity
Lin et al., 2021 [154]	206/206 (100%)	22C3 (P)	CPS score ≥ 1
Imamura et al., 2021 [18]	35/161 (22%)	NR	NR
Petrylak et al., 2021 [17]	0/33 (0%)	SP142 (V)	≥5%
Sun et al., 2021 [13]	0/48 (0%)	22C3 (MM, D)	>1%
Shim et al., 2021 [12]	71/171 (42%) (m) 122/171 (71%) (n)	22C3 (D)	Any positivity
Vardaki et al., 2021 [11]	1/1 (100%)	RM-320 (RM, R, 1:300)	NR
Sharma et al., 2020 [8]	16/63 (25%)	28-8 (RM, P)	≥1%
Ross et al., 2020 [32]	0/2 (0%)	22C3 (MM, Ag)	Semiquantitive (negative: 0%)
Antonarakis et al., 2020 [9]	156/258 (60%)	22C3 (MM, Ag)	CPS score ≥ 1
Graff et al., 2020 [21]; Graff et al., 2016 [90]	0/28 (0%)	E1L3N (RM, CS) 22C3 (MM, Ag)	NR
Sharma et al., 2020 [27]; Sharma et al., 2019 [35]	29/220 (13%)	22C3 (MM, Ag)	mo/st ≥ 1% or we ≥ 10%
Liu et al., 2020 [112]	2/165 (1%) (1 we, 1 mo)	28-8 (D) E1L3N (RM, CS)	NR
Obradovic et al., 2020 [29]	0/29 (0%)	SP263 (RM, V, 1:300)	≥1% (any intensity)
Shaw et al., 2020 [36]	20/91 (22%)	NR	≥1% (m)
Zhou et al., 2019 [120]	47/122 (39%)	PA5-18337 (GP, T, 1:1000)	Low (<median) vs. high (>median): % positive cells (0: <5%; 1: 6–25%; 2: 25–50%; 3: >50%) × intensity (0: 0; 1: we; 2: mo; 3: st).
Matveev et al., 2019 [37] (°)	14/45 (31%)	NA	≥1% (≥5% hyperexpression)
Matveev et al., 2019 [38]	10/35 (29%)	28-8 (RM, A)	≥1%
Iacovelli et al., 2019 [39]	15/32 (47%)	28-8 (RM, P)	≥1%
Lindh et al., 2019 [41]	2/42 (5%)	SP263 (RM, V)	≥1%
Xian et al., 2019 [43]	50/279 (18%) (≥2+: 6/279, 2%)	E1L3N (RM, CS, 1:240)	≥1% (m, c) (1+: 1–5%; 2+: 5–25%; 3+: 25–50%; 4+: 50–100%)
Li et al., 2019 [44]	63/127 (50%)	ABM4E54 (MM, A, 1:200)	Low (<median) vs. high (>median): % positive cells (0–100%) × dominant intensity grade (0, absent; 1, we; 2, mo; 3, st)
Mo et al., 2019 [50]	1/80 (1%)	E1L3N (1:200), E1J2J (1:100) (RM, CS)	NR (m)
Jin et al., 2019 [53]	65/145 (45%)	E1L3N (RM, CS) anti-PD-L1 (RP, Pr)	NR
Papanicolau-Sengos et al., 2019 [51]	1/19 (5%)	22C3 (I)	NR
Li et al., 2019 [78]	1/30 (3%)	E1L3N	≥1%
Richardsen et al., 2019 [42]; Ness et al., 2017 [79]	371/402 (92%)	E1L3N (RM, CS)	High score ≥ 1 (at least we) (m, c) (homogenous expression)
Hansen et al., 2018 [62]	12/23 (52%)	NR	≥1%
Richter et al., 2018 [55]	1/24 (4%)	28-8 (RM, A, 1:400)	Semiquantitive; 0: 0%; 1: ≤1%; 2: 1–10%; 3: 10–50%.
Hahn et al., 2018 [57]	1/21 (5%)	SP263 (RM, V)	High: ≥5% Low: <5%
Nava Rodrigues et al., 2018 [59]	9/51 (18%)	E1L3N (RM, CS, 1: 200)	TPS
Wang et al., 2018 [61]	0/21 (0%)	SP263 (RM, V)	≥25%
Haffner et al., 2018 [66]	39/508 (8%)	SP263 (RM, V, 1:300)	≥1%
Nagaputra et al., 2018 [67]	45/211 (21%)	NR	≥1%
Karzai et al., 2018 [54]	2/5 (40%)	E1L3N (CS, 1:50)	≥1%
Fankhauser et al., 2017 [74]	5/82 (6%)	E1L3N (RM, CS); SP263 (RM, V)	>1%
Calagua et al., 2017 [75]	21/177 (12%): 18/130 (14%) (hormone-naïve); 3/44 (7%) (AAPL)	E1L3N (RM, CS, 1:200) SP142 (RM, SPB, 1:100).	≥1% (mo, st)
Petitprez et al., 2017 [77]	7/51 (14%)	22C3 (MM, Ag)	≥1%
Baas et al., 2017 [80]	Low: 23/25 (92%) High: 2/25 (8%)	22C3 (MM, Ag)	Semiquantitative 0 to 5 scale(subjective score) (0–2: low; 3–5: high)
Tretiakova et al., 2017 [83]	1/127 (1%)	22C3, 28.8, E1L3N, SP142	NR
Najjar et al., 2017 [84]	7/129 (24%)	SP142 (RM, SPB)	≥1% (high ≥25%) (m)
Hashimoto et al., 2016 [85]	2/110 (2%)	SP142 (RM, SPB)	NR
Gevensleben et al., 2016 [93]; Goltz et al., 2016 [89]	High: 486/820 (59%)	EPR1161(2) (A, 1:75)	Semiquantitative (m and/or c)High: >median value
Massari et al., 2016 [92]	7/15 (47%)	Anti-PD-L1 (RM, SB)	1+(we)/2+ (st) in >50% cells (m)
Martin et al., 2015 [94]	11/20 (55%)	5H1 (MM)	>5% (m)
Taube et al., 2014 [98,99]	0/2 (0%)	5H1 (MM)	≥5% (m)
Topalian et al., 2012 [100]	0/2 (0%)	5H1 (MM)	≥5%

(°) only the abstract was available; A: Abcam, Cambridge, UK; AAPL: abiraterone acetate, prednisone, and leuprolide; Ag: Agilent Technologies, Santa Clara, CA, USA; c: cytoplasmic; CPS: combined positive score (number of PD-L1+ cells, including tumor cells, lymphocytes, and macrophages, divided by the total number of tumor cells, × 100); CS: Cell Signaling Technology, Danvers, MA, USA; D: Dako, Agilent Technologies, Santa Clara, CA, USA; GP: goat polyclonal; I: Immune Report Card^®^ and OmniSeq Comprehensive^®^ assays (OmniSeq, Inc., Buffalo, NY, USA); m: membranous; MM: mouse monoclonal; mo: moderate; n: nuclear; NA: not available; NR: not reported; P: pharmDx, Agilent Technologies, Santa Clara, CA, USA; Pr: Proteintech Group, Rosemont, IL, USA; R: RevMAb Biosciences, San Francisco, CA, USA; RM: rabbit monoclonal; RP: rabbit polyclonal; SB: Sino Biological, PA, USA; SPB: Spring Biosciences, Pleasanton, CA, USA; st: strong; T: Thermo Fisher Scientific, Inc., MA, USA; TPS: tumor proportion score (number of positive tumor cells/total number of viable tumor cells); V: Ventana Medical Systems, Tucson, AZ, USA; we: weak.

**Table 2:** PD-L1 antibody clones: positivity rate in tumor cells.

Clone	PD-L1 Positivity Rate	References
22C3	473/1155 (41%) ° 111/691 (11%) *	[9,12,13,21,27,32,35,51,77,80,83,90,154]
SP263	45/703 (6%)	[29,41,57,61,66,74]
PA5-18337	47/122 (39%)	[120]
28-8 (#)	43/281 (15%)	[8,38,39,55,83]
E1L3N (#, $, @, §)	547/1579 (35%)	[21,42,43,50,53,54,59,74,75,78,79,83,90]
ABM4E54	63/127 (50%)	[44]
SP142 (§)	10/399 (3%)	[17,83,84,85]
Anti-PD-L1 (@)	7/15 (47%)	[92]
5H1	11/24 (46%)	[94,98,99,100]
RM-320	1/1 (100%)	[11]

°: including combined proportion score ≥1 cases. *: excluding combined proportion score ≥1 cases. (#): in a series [112], it was unclear how many cases were tested for clones 28-8 and/or E1L3N: they were not counted. ($): it was unclear if the 1/80 E1L3N + case of Mo et al. [50] was also positive for clone E1J2J. (@): in the series of Jin et al. [53], it was unclear how many E1L3N + cases (65/145) were also positive for anti-PD-L1 clone (Rabbit polyclonal, Proteintech Group, IL, US). (§): in the series of Calagua et al. [75], it was unclear how many E1L3N-positive cases (21/177) were also positive for clone SP-142.

## Supplementary Materials

The following Tables are available online at https://www.mdpi.com/article/10.3390/cells10113166/s1. Table S1: Details of the cases tested by immunohistochemistry. Table S2: Evaluation of PD-L1 expression density in tumor tissue.
