# Supplementary material for: What Do We Have to Know about PD-L1 Expression in Prostate Cancer? A Systematic Literature Review. Part 1: Focus on Immunohistochemical Results with Discussion of Pre-Analytical and Interpretation Variables
Source: Cells. 2021 Nov 14;10(11):3166. doi: 10.3390/cells10113166 (PMC8625301; doi:10.3390/cells10113166)
Supplement: Supplementary file 1 [file cells-10-03166-s001.zip › cells-1398278-supplementary.pdf]

cells

Review

What Do We Have to Know about PD-L1 Expression in Prostate Cancer? A Systematic Literature review. Part 1: Focus on Immunohistochemical Results with Discussion of Pre-Analytical and Interpretation Variables

Andrea Palicelli [1,*], Martina Bonacini [2], Stefania Croci [2], Cristina Magi-Galluzzi [3], Sofia Cañete-Portillo [3], Alcides Chaux [4], Alessandra Bisagni [1], Eleonora Zanetti [1], Dario De Biase [5], Beatrice Melli [6,7], Francesca Sanguedolce [8], Moira Ragazzi [1], Maria Paola Bonasoni [1], Alessandra Soriano [9,10], Stefano Ascani [11,12], Maurizio Zizzo [13], Carolina Castro Ruiz [7,13], Antonio De Leo [14], Guido Giordano [15], Matteo Landriscina [15], Giuseppe Carrieri [16], Luigi Cormio [16], Daniel M. Berney [17], Daniel Athanazio [18], Jatin Gandhi [19], Alberto Cavazza [1], Giacomo Santandrea [1,7], Alessandro Tafuni [1,20] and Magda Zanelli [1]

Table S1. Details of the cases tested by immunohistochemistry.

Authors	PD-L1 positivity rate	Details
**Lin et al., 2021 [154]**	206/206 (100%)	ST: NR<br>GG: variable<br>Stage: mCRPC; pT2-3 M0-1<br>Treatment: no previus treatment; PEM + ENZ or PEM alone
**Imamura et al., 2021 [18]**	35/161 (22%)	ST: RP<br>GG: NR<br>Stage: pT2-3<br>Treatment: 6 CRPC
**Petrylak et al., 2021 [17]**	0/33 (0%)	ST: NR<br>GG: variable<br>Stage/Treatment: Atezolizumab to mCRPC (22 with ≥3 prior lines of therapy; 32 prior ENZ; 13 prior sipuleucel-T)
**Sun et al., 2021 [13]**	0/48 (0%)	ST/GG/Stage: NR<br>Treatment: 18 CRPC
**Shim et al., 2021 [12]**	71/171 (42%) (membranous)<br>122/171 (71%) (nuclear)	ST: biopsy<br>GG: variable<br>Stage: clinically localized and locally advanced PC<br>Treatment: primary RT
**Vardaki et al., 2021 [11]**	1/1 (100%)	ST: biopsy<br>GG: variable<br>Stage: M1<br>NT: NR<br>AT: variable, including Radium-223
**Sharma et al., 2020 [8]**	16/63 (25%)	ST: RP or biopsies<br>GG: variable<br>Stage: M0 -1

		NT: NR<br>AT: ADT (GnRH analogue or bilateral orchiectomy) + Nivolumab + Ipilimumab to CRPC
**Ross et al., 2020 [32]**	0/2 (0%)	ST: biopsies<br>GG: 2-5<br>Stage: pT1c-3 N0-1 M1<br>Treatment: DEG + PEM + WPC to hormone sensitive PC
**Antonarakis et al., 2020 [9]**	156/258 (60%)	ST: biopsies<br>GG: variable<br>Stage/NT/AT: PEM to metastatic or locally confined CRPC
**Graff et al., 2020 [21]**<br>**Graff et al., 2016 [90]**	0/28 (0%)	ST: biopsies<br>GG: 1-5<br>Stage: pT1c-3 N0-1 M1<br>NT: NR<br>AT: ENZ + PEM to CRPC
**Sharma et al., 2020 [27]; Sharma et al., 2019 [35]**	29/220 (13%)	ST: RP (TMA)<br>GG: 1-5<br>Stage: pT2-3ab<br>NT: NR<br>AT: hormone-sensitive; Short-term ADT (DEG) + PEM + WPC
**Liu et al., 2020 [112]**	2/165 (1%) (1 we, 1 mo)	ST: RP (TMA)<br>GG/Stage/Treatment: NR
**Obradovic et al., 2020 [29]**	0/29 (0%)	ST: RP (TMA)<br>GG: ≥ 3<br>Stage: pT1c-T3b IR/HR<br>NT: no<br>AT: (DEG) vs (Cy/GVAX + DEG) 1:1 randomization
**Shaw et al., 2020 [36]**	20/91 (22%)	ST: RP, biopsy<br>GG/Stage/NT/AT: 50 HR; 41 MP
**Zhou et al., 2019 [120]**	47/122 (39%)	ST: RP (TMA)<br>GG: 1-5;<br>Stage: pT2-4<br>NT: no<br>AT: NR
**Matveev et al., 2019 [37]**(°)	14/45 (31%)	ST: RP<br>GG/Stage/NT/AT: NA
**Matveev et al., 2019 [38]**	10/35 (29%)	ST/GG/Stage: NR<br>NT: no<br>AT: ADT (MTS) (mCRPC)
**Iacovelli et al., 2019 [39]**	15/32 (47%)	ST: TMA<br>GG: various; Stage: N1±M1<br>NT: NR

		AT: mCS; ADT (32); Do (%)
**Lindh et al., 2019 [41]**	2/42 (5%)	ST: RP, TURP (TMA)<br>GG: 2-5<br>Stage/NT/AT: NR
**Xian et al., 2019 [43]**	50/279 (18%) (6/279 ≥2+)	ST: RP<br>GG: 1-5<br>Stage: pT1-4 N0-1<br>NT/AT: NR
**Li et al., 2019 [44]**	63/127 (50%)	ST: RP<br>GG: 1-5<br>Stage: HR (pT ≥3, pN ≥1, positive surgical margins) or (pT≤2, GG ≥4, PSA ≥20 ng/ml)<br>NT: NR<br>AT: ADT [LHRHA +/- anti-androgen (bicalutamide)]
**Mo et al., 2019 [50]**	1/80 (1%)	ST: RP (RLPC); TURP (CRPC)<br>GG: ≥1<br>Stage: variable<br>NT/AT: NR (73 RLPC, 7 CRPC)
**Jin et al., 2019 [53]**	65/145 (45%)	ST: TMA (§)<br>GG: NR<br>Stage: pM1<br>NT/AT: NR
**Papanicolau-Sengos et al., 2019 [51]**	1/19 (5%)	ST: RP (11), MTS (8)<br>GG: 2-5<br>Stage: pN1 or pM1<br>NT: NR<br>AT: androgen therapy (9 CRPC, 10 CSPC)
**Li et al., 2019**	1/30 (3%)	ST: NR (TMA)<br>GG: high<br>Stage/NT/AT: NR
**Richardsen et al., 2019 [42]; Ness et al., 2017 [79]**	371/402 (92%)	ST: RP<br>GG: 1-5<br>Stage: pT2-3b<br>NT/AT: NR
**Hansen et al., 2018 [62]**	12/23 (52%)	ST: biopsy<br>GG: NR<br>Stage: metastatic or locally advanced<br>NT/AT: PEM to CRPC
**Richter et al., 2018 [55]**	1/24 (4%)	ST: biopsies, RP, TURP<br>GG: 1-5<br>Stage: N1 or M1<br>NT: NR

		AT: mCRPC; ChT (1-2 lines) + ENZ (#)
**Hahn et al., 2018 [57]**	1/21 (5%)	ST: RP<br>GG:1-5<br>Stage: pT2-3b N0-1<br>NT: no<br>AT: variable (3 CRPC)
**Nava Rodrigues et al., 2018 [59]**	9/51 (18%)	ST: RP, biopsy, TURP<br>GG/Stage: variable<br>NT: NR<br>AT: RT (some)
**Wang et al., 2018 [61]**	0/21 (0%)	ST: RP<br>GG: 1-5<br>Stage/AT: NR<br>NT: no
**Haffner et al., 2018 [66]**	39/508 (8%)	ST: RP, biopsy, autopsy (TMA)<br>GG:1-5<br>Stage: pT2-4 N0-1<br>NT: NR<br>AT: ADT in some cases (57 mCRPC)
**Nagaputra et al., 2018 [67]**	45/211 (21%)	ST: RP (TMA)<br>GG/Stage/NT/AT: NR
**Karzai et al., 2018 [54]**	2/5 (40%)	ST/GG: NR<br>Stage: metastatic<br>NT/AT: mCRPC previously treated with ENZ and/or abiraterone; patients received durvalumab + olaparib
**Fankhauser et al., 2017 [74]**	5/82 (6%)	ST: TMA (TURP, RP, MTS)<br>GG: NR<br>Stage: 96 localized, also pN1/M1<br>NT: NR<br>AT: LHRH agonists (goserelin, leuprorelin), anti- androgens (bicalutamide) (82 CRPC), ChT (Do) or RT.
**Calagua et al., 2017 [75]**	21/177 (12%):<br>18/130 (14%) (hormone-naïve);<br>3/44 (7%) (AAPL)	ST: RP<br>GG:1-5<br>Stage: pT2/3b Nx/0/1<br>NT: 44 AAPL<br>AT: NR
**Petitprez et al., 2017 [77]**	7/51 (14%)	ST: RT, pelvic LND<br>GG: 1-5<br>Stage: pT2-3b N1 M0<br>NT: no<br>AT: RT (some)

**Baas et al., 2017 [80]**	Low: 23/25 (92%)<br>High: 2/25 (8%)	ST: RP, biopsy<br>GG: 4-5<br>Stage/NT/AT: NR
**Tretiakova et al., 2017 [83]**	1/127 (1%)	ST: NR (TMA)<br>GG/Stage/NT/AT: NR
**Najjar et al., 2017 [84]**	7/129 (24%)	ST/GG/Stage/NT/AT: NR
**Hashimoto et al., 2016 [85]**	2/110 (2%)	ST: RP (103), biopsy (7)<br>GG/Stage/NT/AT: NR
**Gevensleben et al., 2016 [93]; Goltz et al., 2016 [89]**	High: 486/820 (59%)	ST: RP (TMA)<br>GG: 1-5<br>Stage: pT2-4Nx/0/1
**Massari et al., 2016 [92]**	7/15 (47%)	ST: RP (TMA)<br>GG: 2-5<br>Stage/NT: NR<br>AT: HT (15 CRPC); RT (7)
**Martin et al., 2015 [94]**	11/20 (55%)	ST: RP<br>GG/Stage: NR<br>NT: leuprolide (11)<br>AT: NR
**Taube et al., 2014 [98,99]**	0/2 (0%)	ST/GG/Stage/NT: NR<br>AT: CRPC
**Topalian et al., 2012 [100]**	0/2 (0%)	ST/GG/Stage: NR<br>Treatment: anti-PD-1 antibody to CRPC

(°): only abstract available; (§): 145 tissue microarray cores from 53 samples of metastatic castration-resistant prostate cancer (20 bone metastases, 33 non-bone metastases); (#): Enzalutamide standard dose (160 mg orally/die, as four 40 mg capsules).

AAPL: Abiraterone Acetate + Prednisone and Leuprolide; ADT: androgen deprivation therapy; AT: adjuvant therapy; ChT: chemotherapy; CRPC: castration-resistant prostate cancer; CSPC: castration-sensitive prostate cancer; Cy/GVAX: cyclophosphamide 200 mg/m2 intravenously and GVAX (2.5×108 PC3 cells, 1.6×108 LNCaP cells) given 2 weeks before Degarelix; DEG: Degarelix; Do: docetaxel; ENZ: enzalutamide; GG: Grade Group; GnRH: gonadotropin releasing hormone; HR: high-risk prostate cancer; HT: hormone-therapy; IR: intermediate-risk prostate cancer; LHRHA: luteinizing hormone releasing hormone analogue; LND: lymph nodes; mCS: metastatic castration-sensitive prostatic adenocarcinomas; mCRPC: metastatic castration-resistant prostate cancer; MP: metastatic prostatic cancer; MTS: metastases; NA: not available; NR: not reported; NT: neoajuvant therapy; PC: prostate cancer; PEM: pembrolizumab; RLPC: regionally localized prostate cancer; RP: radical prostatectomy; RT: radiation therapy; ST: sample type; TMA: tissue microarray; TURP: transurethral resection of the prostate; WPC: whole-prostate cryoablation.

Table S2. Evaluation of PD-L1 expression density in tumor tissue.

Authors	Samples	Clinic-pathologic notes	Materials and methods -Details	Clinical outcome
**Brady et al., 2021 [15]**	27	ST: biopsy (metastases)<br>GG: NR<br>Stage: M1<br>Treatment: CRPC	Digital spatial gene expression profiling; ROI	PD-L1 protein levels were not detectable above background in any tumor or tumor ROI. The expression of CTLA4 and PD-1, were similarly below measurable levels in >90% of ROIs, either by transcript or antibody-based measurements.
**Sater et al., 2020 [34]**	26	ST: RP, biopsies<br>GG: 1-5<br>Stage: cIA-IIB<br>NT: PROSTVAC<br>AT: NR	Immunofluorescence; ROI (mm^2, original magnification 40x, 0.25μm/pixel resolution)	Neoadjuvant PROSTVAC can induce both tumor immune response and peripheral immune response. Heterogeneity of the immune infiltrate in both cell subtypes and compartmental distribution.<br>3/26 (11.5%): focal PD-L1 + (RP)<br>0/26 (0%): PD-L1 + (biopsies)
**Vicier et al., 2020 [25]**	109	ST: RP (TMA)<br>GG: 1-5<br>Stage: T2-4N0<br>NT: NR<br>AT: RT (n:37) ADT (n:11)	Fluorescent immunohistochemistry (clone E1L3N, rabbit monoclonal, Cell Signaling Technology, Danvers, MA, US, dilution 1:200); digital analysis (Halo Image Analysis platform, Indica Labs, Albuquerque, NM, US); Quantitative multispectral imaging analysis; Total density (number of positive cells divided by the combined glandular and stromal area) was dichotomized by the median as "high" (>median) or "low" (≤median).	While neither low CD8 or high PD-L1 alone were independent predictors of BCR or MFS on multivariable analysis, men with low CD8 and/or high PD-L1 had a significantly shorter time to BCR (median 3.5 years vs. NR) and MFS (median 10.8 vs. 18.4 years) compared to those with high CD8 and low PD-L1 expression..
**Ryan et al., 2020 [26]**	8	ST: RP<br>GG: 1-5<br>Stage: pT2-pT3ab<br>NT: rituximab<br>AT: NR	Immunohistochemistry (clone E1L3N, Rabbit monoclonal, Cell Signaling Technology, Danvers, MA, US), Spectrum Analysis algorithm package; ImageScope analysis software (Aperio Technologies, Inc., Vista, CA, US); PD-L1 density: stained area(mm^2)/total area (mm^2)	PD-L1 staining primarily occurred in tertiary lymphoid structures. Non-significant decrease in the mean PD-L1 density in the rituximab-treated tissue vs control samples (p=0.36).
**Wagle et al., 2020 [28]**	180	ST: RP<br>GG: 1-5<br>Stage: I-IV<br>(32 early/intermediate: pT1a-pT2c; 123 late: pT3a-pT4b; 15 metastatic to lymph nodes, lungs, bone, or bladder)<br>NT: NR<br>AT: variable	Immunohistochemistry (clone SP142, Ventana Medical Systems, Tucson, AZ, US). Digital analysis: VIS software (version 6.6.1, Visiopharm, Denmark).<br>Relative area estimate (% of tumor area covered by PD-L1+ cells) (scoring as in the Ventana SP142 PD-L1 immunohistochemical assay brochure)	High tumor fusion burden (number of fusions/10,000 genes) correlated with high immune infiltration, PD-L1 expression on immune cells (negative on tumor cells), and immune signatures representing activation of T-cells and M1 macrophages, while it inversely correlated with immune suppressive signatures. Only late metastatic samples (n: 3) showed ≥10% of PD-L1+ inflammatory cells.
**Ihle et al., 2019 [31]**	15	ST: bone metastases (10 lytic, 5 blastic)<br>GG: NR<br>Stage: IV	Digital counting on ROI (nCounter; counts processor: Digital Space Profiling App v5.3)	PD-L1-expression was higher in blastic metastases (vs lytic)

		NT: NR<br>AT: various (hormonal, RT, ChT)		
**Kazantseva et al., 2019 [40]**	122	ST: RP or biopsy<br>GG: 1-5<br>Stage: NR<br>NT: NR<br>AT: NR	Immunohistochemistry, immunofluorescence; clone CAL10 (Biocare Medical, Pacheco, CA, US); Aperio Membrane Algorithm, Aperio, Vista, CA, US; Total amount of positive tumor + immune cells in 30 high power fields.	3 different groups defined by clustering analysis. The PD-L1 positive cells were significantly higher in Group A (n: 43, 35%) (median = 72; 95% confidence interval: 42–120) compared to Groups B (n: 50, 41%) and C (n: 29, 24%) (median 30; 95% confidence interval 17–53; p = 0.01). Group A also had higher Δ133TP53 and TP53β, significantly higher number of infiltrating CD3+ T-cells, CD4+ T-cells, CD8+ T-cells and CD20+ B-cells, and increased numbers of PD-1-positive T-cells (compared to Groups B,C) significantly higher numbers of infiltrating CD163+ macrophages (compared to Group C), higher Ki67 index (compared to Group B).
**Scimeca et al., 2019 [48]**	50 (+ 50 BPH)	ST: RP (TMA)<br>GG: 1-5<br>Stage: NR<br>NT: no<br>AT: NR	Immunohistochemistry; clone QR1, rabbit monoclonal, Quartett, Berlin, Germany; number of positive cells/9.42 mm^2 (± standard error of mean)	Significant increase of PD-L1+ ($p = 0.0047$) and PTX3+ ($p < 0.0001$) cells in PCs respect to benign lesions. Inflammatory infiltrate of PD-L1+ PCs showed decrease of PD-1+ lymphocytes and tumor-infiltrated macrophages (mainly M2 subpopulation). PTX3 expression showed an inverse correlation with the number of PD-L1+ PC-cells.

ADT: androgen deprivation therapy; AT: adjuvant therapy; BCR: biochemical recurrence; BPH: benign prostatic hyperplasia; ChT: chemotherapy; CRPC: castration-resistant prostate cancer; GG: Grade Group; MFS: metastasis-free survival; NR: not reported; NT: neoadjuvant treatment; PC: prostatic adenocarcinoma; PROSTVAC: recombinant vaccinia (rV)-PSA(L155)- TRICOM (2×10^8 IU subcutaneously), followed by monthly boosts on weeks 5, 9 and 13 with recombinant fowlpox (rF)-PSA(L155)-; ROI: regions of interest; RP: radical prostatectomy; RT: radiotherapy; ST: specimen type; TMA: tissue microarray.
